# Modifying effect of the serum level of brain-derived neurotrophic factor (BDNF) on the association between *BDNF* methylation and long-term cardiovascular outcomes in patients with acute coronary syndrome

**DOI:** 10.3389/fcvm.2022.1084834

**Published:** 2023-01-18

**Authors:** Wonsuk Choi, Hee-Ju Kang, Ju-Wan Kim, Hee Kyung Kim, Ho-Cheol Kang, Sung-Wan Kim, Jung-Chul Kim, Youngkeun Ahn, Myung Ho Jeong, Jae-Min Kim

**Affiliations:** ^1^Department of Internal Medicine, Chonnam National University Hwasun Hospital, Chonnam National University Medical School, Hwasun, Republic of Korea; ^2^Department of Psychiatry, Chonnam National University Medical School, Gwangju, Republic of Korea; ^3^Department of Surgery, Chonnam National University Medical School and Hospital, Gwangju, Republic of Korea; ^4^Department of Cardiology, Chonnam National University Medical School, Gwangju, Republic of Korea

**Keywords:** brain derived neurotrophic factor (BDNF), BDNF methylation, acute coronary syndrome (ACS), outcome, biomarker

## Abstract

**Introduction:**

This study investigated the potential modifying effects of the serum brain-derived neurotrophic factor (sBDNF) level on the association between *BDNF* methylation status and long-term cardiovascular outcomes in acute coronary syndrome (ACS) patients.

**Methods:**

From 2006 to 2012, hospitalized ACS patients were consecutively recruited. The sBDNF level and *BDNF* methylation status were assessed at baseline in 969 patients who were followed up for major adverse cardiac events (MACEs) over 5–12 years, until 2017 or death. Cox proportional hazards models were utilized to compare the time to first composite or individual MACEs between individuals with lower and those with higher average *BDNF* methylation levels in the low and high sBDNF groups, respectively. The modifying effects of the sBDNF and average *BDNF* methylation levels on first composite and individual MACEs were analyzed using Cox proportional hazards models after adjusting for potential covariates.

**Results:**

In the low sBDNF group, a higher average *BDNF* methylation level was linked to an increase in composite MACEs independent of confounding variables, but not in the high sBDNF group [HR (95 percent CI) = 1.04 (0.76–1.44)]. The interaction effect between the sBDNF and average *BDNF* methylation levels on composite MACEs was significant after adjusting for covariates (*P* = 0.008).

**Conclusion:**

Combining the *BDNF* methylation status and sBDNF levels may help identify ACS patients who are likely to have unfavorable clinical outcomes.

## Introduction

Neurotrophins are involved in the development, maintenance, and plasticity of neurons (1), as well as in the development of the heart and blood vessels (2–4). Among the neurotrophins, brain-derived neurotrophic factor (BDNF), a crucial mediator of homeostasis and pathogenesis in the cardiovascular system, has garnered considerable attention (5).

Previous studies have reported a relationship between altered BDNF signaling pathways and CVD. Patients with acute coronary syndrome (ACS) showed reduced circulating levels of BDNF compared with control subjects in a cross-sectional study (6). Higher circulating BDNF levels were longitudinally related to a lower risk of CVD and mortality in a general population cohort (7). As in the general population, lower circulating BDNF levels negatively affected clinical outcomes in patients with angina pectoris (8) or heart failure (9).

A decrease in CpG methylation within the regulatory region of the *Bdnf* gene has been linked with increased production of BDNF in neurons (10). Given the findings of prior clinical studies exploring the link between BDNF and CVD (6–9), it is likely that *BDNF* hypermethylation is linked to the development of CVD and a poor prognosis. In our previous study, a higher *BDNF* methylation level was associated with composite major adverse cardiovascular events (MACEs) in ACS patients (11). Since sBDNF and *BDNF* methylation levels have been linked to cardiovascular outcomes in previous research (8, 9, 11), and there is a potential link between sBDNF and *BDNF* methylation levels (10), it is anticipated that these two may interact to affect cardiovascular outcomes. However, no research has been done on this matter.

Using information from a prospective study of ACS patients in Korea, we examined the modifying effect of the serum BDNF (sBDNF) level on the association between *BDNF* methylation and long-term cardiovascular outcomes.

## Methods

### Study overview and participants

All analyses employed data from the Korean DEPression in ACS (K-DEPACS) study, which used a naturalistic prospective design to explore the psychological consequences of ACS (12). Supplementary Figure S1 shows a summary of the current study as well as the approach used for recruiting participants. From 2006 to 2012, ACS patients hospitalized at the Department of Cardiology of Chonnam National University Hospital in Gwangju, South Korea who satisfied the eligibility criteria (Supplementary material) were recruited consecutively. The Korean Circulation Society proposed this department in 2005 as the key coordination hub for the Korea Acute Myocardial Infarction Registry (KAMIR) (13). KAMIR is a registry created as a surveillance platform to monitor clinical outcomes of patients with acute MI without exclusion criteria to reflect real-world practice; this enables prospective associations to be assessed for a variety of exposures or interventions with long-term cardiac outcomes. The research cardiologists managed patients' ACS in accordance with global standards (14). Patients who met the inclusion criteria and agreed to participate in the study were assessed as inpatients for baseline testing within 2 weeks (mean ± standard deviation: 6.3 ± 2.4 days) of ACS occurrence. The baseline sample comprised these patients who agreed to blood drawing. The cardiovascular outcomes of all participants were followed until 2017 or death. The Institutional Review Board of Chonnam National University Hospital approved this study (CNUH I-2008-02-027). The consent form was reviewed by all participants, and written informed consent was obtained.

### Primary measures

#### sBDNF level

Before blood collection, participants were told to fast the previous night (apart from water). Following that, they were instructed to stay still and unwind for 25–45 min before blood samples were taken. Serum was prepared in a room temperature. The Quantikine^®^ ELISA Human BDNF Immunoassay (R&D Systems, Inc., Minneapolis, MN, USA) was used to assess the sBDNF level at the Global Clinical Central Lab (Yongin, Korea). Patients were divided into two groups for the initial analysis: those with low sBDNF and those with high sBDNF levels (based on the median value). The sBDNF level was analyzed as a continuous variable in later analyses.

#### BDNF methylation status

Standard techniques were used to extract DNA from venous blood. The promoter region of *BDNF* exon VI, which also contains a CpG-rich area with nine CpG sites, was chosen for the methylation investigation. This region is placed at nucleotides −612 to −463 relative to the transcriptional start site in exon VIII (Supplementary Figure S3). More information on *BDNF* methylation is provided in the Supplementary material. According to prior research (15), the average percentage of *BDNF* methylation was classed as a binary variable with the value “lower (<38.50)” or “higher (≥38.50).” Since there is no absolute cutoff value for *BDNF* methylation, and maximizing the number of patients in both groups increases the statistical power of the analysis of the effect of *BDNF* methylation on the outcome variables, we divided the two groups based on the median average *BDNF* methylation value. The average percentage of *BDNF* methylation was analyzed as a continuous variable in subsequent analyses.

### Baseline covariates

Covariates that potentially affect cardiovascular outcomes were examined within 2 weeks of ACS occurrence. Throughout the evaluation, data on age, sex, years of education, living status (living alone or not), type of residence (owned or rented), and current occupation (employed or not) were collected. Fasting glucose, total cholesterol, BUN, and creatinine levels were assessed using the Hitachi Automatic Analyzer 7,600 (Hitachi, Tokyo, Japan). Personal and family histories of depression, as well as the Beck Depression Inventory score (16), were used to evaluate depression status in the participants. Personal and family histories of ACS, diagnosed diabetes, diagnosed hypertension, hypercholesterolemia based on the fasting serum total cholesterol level (>200 mg/dL) or a history of hyperlipidemia with ongoing treatment, obesity based on measured body mass index (BMI > 25 kg/m^2^), and a reported current smoking status were all used to assess cardiometabolic risk factors. The Killip classification (17) was used to assess current cardiac status, and LVEF was calculated using echocardiography. Two cardiac enzymes, troponin I and creatine kinase (CK)-MB, were also examined.

### Outcomes

As the primary outcome, a MACE was defined as the composite of all-cause mortality, myocardial infarction (MI), and percutaneous coronary intervention (PCI). Secondary outcomes were all-cause mortality, cardiac death (defined as sudden death for no apparent reason, death from arrhythmias, MI, or heart failure, or death due to heart surgery or endocarditis), MI, and PCI. An independent endpoint committee made up of study cardiologists decided on all potential events. Detailed information on long-term cardiovascular outcome is provided in the Supplementary material.

### Statistical analysis

The baseline data was compared according to the sBDNF level (low vs. high) using the independent *t*-test or chi-square test. The covariates used in the adjusted analyses were chosen based on a data generation system and the variables' propensity for collinearity (18). The correlation between the baseline sBDNF level and average *BDNF* methylation level was analyzed by Spearman rank-order correlation analysis. The cumulative proportion of participants having composite or individual MACEs (defined by the date of the first incident for each patient) was compared between those with lower and those with higher average *BDNF* methylation levels in the low and high sBDNF groups, respectively, using Kaplan–Meier analysis. Cox proportional hazards models were used to compare the time to first composite or individual MACEs, after adjustment for potential covariates, between individuals with lower and those with higher average *BDNF* methylation values in the low and high sBDNF groups, respectively. The interaction effect between the sBDNF and average *BDNF* methylation levels on first composite or individual MACEs was analyzed using Cox proportional hazards models after adjusting for potential covariates. Schoenfeld residuals tests were carried out to test the proportional hazards assumptions in all models. All statistical tests were two-sided, and statistical significance was determined as a *P*-value < 0.05. IBM SPSS Statistics (version 25) was utilized for the statistical analysis.

## Results

In total 969 (84.1%) of the 1,152 patients examined at baseline gave their consent to offer blood samples (Supplementary Figure S1). Between those who agreed to submit blood samples and those who declined, the baseline values were not substantially different. All participants were followed to assess cardiovascular outcomes for 5–12 years, until 2017 or death [median; mean (standard deviation) duration of follow-up = 8.4; 8.7 (1.5) years].

The levels of sBDNF were 17.6 (9.4) ng/mL for the median (interquartile range), and 17.8 (7.0) ng/mL for the mean (standard deviation). In the 969 study participants, there was no correlation between the baseline sBDNF level and the average *BDNF* methylation level (r^2^ = 0.001, *P* = 0.969). Since the *BDNF* expression is regulated by a genetic polymorphism entailing substitution of valine by methionine at codon 66 (Val66Met) in the pro-BDNF molecule (19), we compared the sBDNF level and *BDNF* methylation according to the presence of BDNF Val66Met polymorphism (Supplementary Table S1). sBDNF level was lower in Val/Met and Met/Met genotype compared to Val/Val genotype. In addition, BDNF methylation was higher in Val/Met genotype compared to Val/Val genotype. Baseline characteristics according to sBDNF level are summarized in Supplementary Table S2. A low sBDNF level was significantly associated with older age, higher frequency of Killip class > 1, and higher CK-MB level. Referencing the system that produced the data and any potential collinearity between the variables (18), 11 parameters (age, sex, Beck Depression Inventory score, depression comorbidity and treatment, previous history of ACS, diabetes, hypertension, hypercholesterolemia, obesity, smoking, and Killip class) were included as covariates in the adjusted analysis [see Supplementary Figure S2 for a directed acylic graph (20–22)].

**Figure 1:**
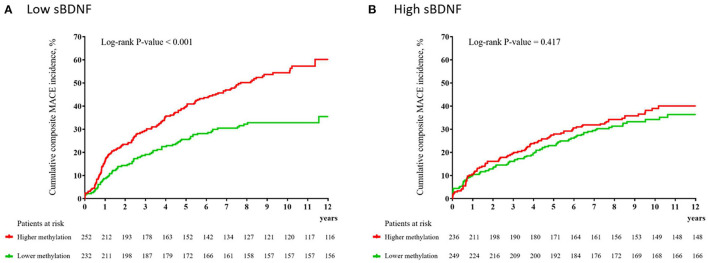
Cumulative incidence (%) of composite major adverse cardiac events (MACEs) according to the average *BDNF* methylation level at baseline in patients with low **(A)** and high **(B)** serum brain-derived neurotrophic factor (sBDNF) levels.

In the low sBDNF group (*n* = 484), the primary outcome (composite MACEs) occurred in 212 participants (43.8%); of the secondary outcomes, all-cause mortality occurred in 104 (21.5%) participants, cardiac death in 59 (12.2%), MI in 55 (11.4), and PCI in 76 (15.7%). In the high sBDNF group (*n* = 485), the primary outcome occurred in 171 participants (35.3%), and the secondary outcome all-cause mortality occurred in 74 (15.3%), cardiac death in 39 (8.0%), MI in 46 (9.5%), and PCI in 63 (13.0%). Figure 1 illustrates the cumulative risk of composite MACEs in subjects with lower vs. higher average *BDNF* methylation levels according to the sBDNF level. In the low sBDNF group, a significant difference was observed: the composite MACE incidence was 32.8% (76/232) in those with lower and 54.0% (136/252) in those with higher methylation levels [log-rank *P*-value < 0.001] (Figure 1A). In addition, significant differences in those with lower vs. higher methylation levels were observed in the incidence of all-cause mortality [15.1% (35/232) vs. 27.4% (69/252), log-rank *P*-value = 0.002] and PCI [12.1% (28/232) vs. 19.0% (48/252), log-rank *P-*value = 0.018] (Supplementary Figure S4). Effects of a higher *BDNF* methylation level on composite MACEs and all-cause mortality were seen in the adjusted analysis (Table 1). However, in the high sBDNF group, significant differences in the primary or secondary outcomes were not observed (Figure 1B, Table 1, and Supplementary Figure S5). In the main analysis, the interaction effect between the sBDNF and average *BDNF* methylation levels on composite MACEs or all-cause mortality was significant after adjusting for covariates (Table 1). When the average *BDNF* methylation level was treated as a continuous variable, the interaction effect between the sBDNF and average *BDNF* methylation level on composite MACEs was also significant (Supplementary Table S3). Similar results were observed when both sBDNF and average *BDNF* methylation levels were treated as continuous variables (Supplementary Table S4). Considering the competing risk situation, we re-analyzed after excluding patients with non-cardiac death before the onset of MI or PCI (Supplementary Table S5) and excluding patients with death before the onset of MI or PCI (Supplementary Table S6), respectively. Generally, similar results were observed to the main analysis. All model assumptions were all met (Schoenfeld *P*-values > 0.3). The sBDNF level had no effect on the incidence of the primary or secondary outcomes in the adjusted analysis (Supplementary Table S7).

**Table 1:** Associations of a higher average BDNF methylation level at baseline with long-term cardiovascular outcomes in patients with ACS according to the sBDNF level.

	**Low sBDNF (*N =* 484)**	**High sBDNF (*N =* 485)**	***P*** **-value for interaction**
Major adverse cardiac events	1.67 (1.25–2.23)^**‡**^	1.04 (0.76–1.44)	0.008
All-cause mortality	1.67 (1.10–2.52)^*^	1.00 (0.61–1.64)	0.044
Cardiac death	1.40 (0.82–2.41)	1.33 (0.67–2.62)	0.539
Myocardial infarction	1.13 (0.65–1.98)	1.41 (0.75–2.66)	0.533
Percutaneous coronary intervention	1.54 (0.96–2.47)	1.02 (0.60–1.72)	0.325

The HR (95% CI) was adjusted for age, sex, Beck Depression Inventory scores, depression comorbidity and treatment, previous history of ACS, diabetes, hypertension, hypercholesterolemia, obesity, smoking, and Killip class.

* *P* < 0.05;

‡ *P* < 0.001.

## Discussion

In this study, we found a modifying effect of the sBDNF level on the association between *BDNF* methylation and long-term cardiovascular outcomes using data from a prospective study of Korean ACS patients. A higher *BDNF* methylation level in patients with low sBDNF levels was a significant predictor of poor long-term cardiovascular outcomes such as composite MACEs, all-cause mortality, and PCI. These results remained reliable after accounting for relevant covariates. However, a higher *BDNF* methylation level had no influence on long-term cardiovascular outcomes in patients with high sBDNF levels.

The methylation status of the *BDNF* genomic region that we evaluated corresponds to an equivalent region in rat *Bdnf*, which was variably methylated and related to *Bdnf* mRNA expression (23, 24). The regulatory region of the *Bdnf* gene's CpG methylation has been shown to be lessened in neurons with increased BDNF synthesis (10). In our previous study, a higher average *BDNF* methylation status was associated with an increased incidence of composite MACEs in ACS patients (11). Because *BDNF* methylation status is linked to neuronal BDNF production, baseline circulating BDNF level may have an impact on these effects. In this study, higher average *BDNF* methylation had a negative impact on long-term cardiac outcomes only in the low sBDNF group. The interaction effect of sBDNF level and average *BDNF* methylation on composite MACE was sustained even when both variables were analyzed as continuous variables. These results might be explained by the synergistic effect of both unfavorable exposures (higher *BDNF* methylation and low sBDNF level). This theory is supported by the fact that higher *BDNF* methylation had less of a negative impact in the high sBDNF group. Furthermore, as this study found no correlation between sBDNF level and *BDNF* methylation, we surmise that the adverse effects of both exposures, which are independent of one another, work in concert.

As mentioned in the Introduction, BDNF stimulates a variety of circulatory system cell types and regulates the growth and dynamics of the cardiovascular microcirculation (25). Based on those results, the relationship between circulating BDNF level and CVD has been investigated. ACS patients showed lower sBDNF levels compared with control subjects in a cross-sectional investigation (6), and a lower sBDNF level was longitudinally associated with CVD incidence and mortality in a general population cohort (7). Furthermore, decreased circulating BDNF levels were linked to a worse clinical outcome in a study of individuals with CVDs such as angina pectoris (8) or heart failure (9). In our study of ACS patients, the sBDNF level, on the other hand, had no effect on long-term cardiovascular outcomes. Differences in the type of underlying disorder could explain why our findings differed from those of previous studies. In our investigation, sBDNF levels were measured within 2 weeks of ACS onset. Because ACS is associated with acute psychosocial stress (26), and the sBDNF level rises in response to acute psychosocial stress (27), the sBDNF level may not have had an effect on long-term cardiovascular outcomes in ACS patients due to overall elevated sBDNF levels. However, because we did not assess the concentration of sBDNF in relation to numerous CVDs in our study, more research is needed to corroborate this.

In interpreting our findings, it is important to take into account a number of study limitations. First, despite this location's prior evaluation (23, 28), the methylation status of just one CpG island in *BDNF* was assessed. Second, only 84% of the baseline sample could undergo methylation analysis due to attrition during the recruitment procedure. However, there were no differences in the baseline demographic and clinical traits of patients with and without access to this information. Third, while the study hypotheses were founded on prior research, the results lacked mechanistic support, demanding additional study. Fourth, although chronic kidney disease is known to be linked to BDNF levels (29) and MACEs (30), this information was not accessible for the study participants. Fifth, it was unclear which tissue is the major source of BDNF detected in the serum. Finally, the study was restricted to one institution, which limits its generalizability but is a benefit because it assures consistency in patient assessment and care.

This study has a number of strengths. It is the first prospective study to look at how sBDNF and average *BDNF* methylation levels interact with regard to long-term cardiovascular outcomes in ACS patients. All eligible patients who had recently had an ACS episode were enrolled as participants at baseline, reducing the likelihood of error caused by varying the testing times and increasing sample homogeneity. All psychiatric and cardiovascular assessments were conducted using well-validated measures. In addition, various covariates were considered in the analyses.

## Conclusions

A higher average *BDNF* methylation status during the acute phase of ACS was associated with worse long-term cardiovascular outcomes only in patients with low sBDNF levels, not in those with high sBDNF levels, regardless of the relevant covariates. These results suggest that combining the *BDNF* methylation and sBDNF levels may help identifying ACS patients who are likely to have unfavorable clinical outcomes. From a therapeutic perspective, patients with *BDNF* hypermethylation and low sBDNF levels require special attention. However, future prospective studies are needed to ascertain whether giving these individuals extra attention results in an improved prognosis for ACS patients.

## Data availability statement

The original contributions presented in the study are included in the article/Supplementary material, further inquiries can be directed to the corresponding author.

## Ethics statement

The studies involving human participants were reviewed and approved by Institutional Review Board of Chonnam National University Hospital. The patients/participants provided their written informed consent to participate in this study.

## Author contributions

WC and J-MK: conceptualization, data curation, formal analysis, and writing. H-JK: data curation and methodology. J-WK: formal analysis and methodology. HK, H-CK, S-WK, J-CK, YA, and MJ: data curation, validation, and project administration. All authors contributed to the article and approved the submitted version.
