# Supplementary material for: Modifying effect of the serum level of brain-derived neurotrophic factor (BDNF) on the association between BDNF methylation and long-term cardiovascular outcomes in patients with acute coronary syndrome
Source: Front Cardiovasc Med. 2023 Jan 18;9:1084834. doi: 10.3389/fcvm.2022.1084834 (PMC9889833; doi:10.3389/fcvm.2022.1084834)
Supplement: Supplementary file 1 [file Table_1.DOCX]

**Supplementary Information**

**Modifying effects of serum brain-derived neurotrophic factor (BDNF) levels on the association between *BDNF* methylation and long-term prognosis of acute coronary syndrome**

Wonsuk Choi^a^, Ju-Wan Kim^b^, Hee-Ju Kang^b^, Hee Kyung Kim^a^, Ho-Cheol Kang^a^, Ju-Yeon Lee^b^, Sung-Wan Kim^b^, Young Joon Hong^c^, Youngkeun Ahn^c^, Myung Ho Jeong^c^, Robert Stewart^d,e^, and Jae-Min Kim^b^

^a^Department of Internal Medicine, Chonnam National University Hwasun Hospital, Chonnam National University Medical School, Hwasun, Korea, ^b^Department of Psychiatry, Chonnam National University Medical School, Gwangju, Korea, ^c^Department of Cardiology, Chonnam National University Medical School, Gwangju, Korea, ^d^ King’s College London, Institute of Psychiatry, Psychology and Neuroscience, London, UK, ^e^ South London and Maudsley NHS Foundation Trust, London, UK

**Supplementary Figure 1**. Flow diagram of the recruitment process.


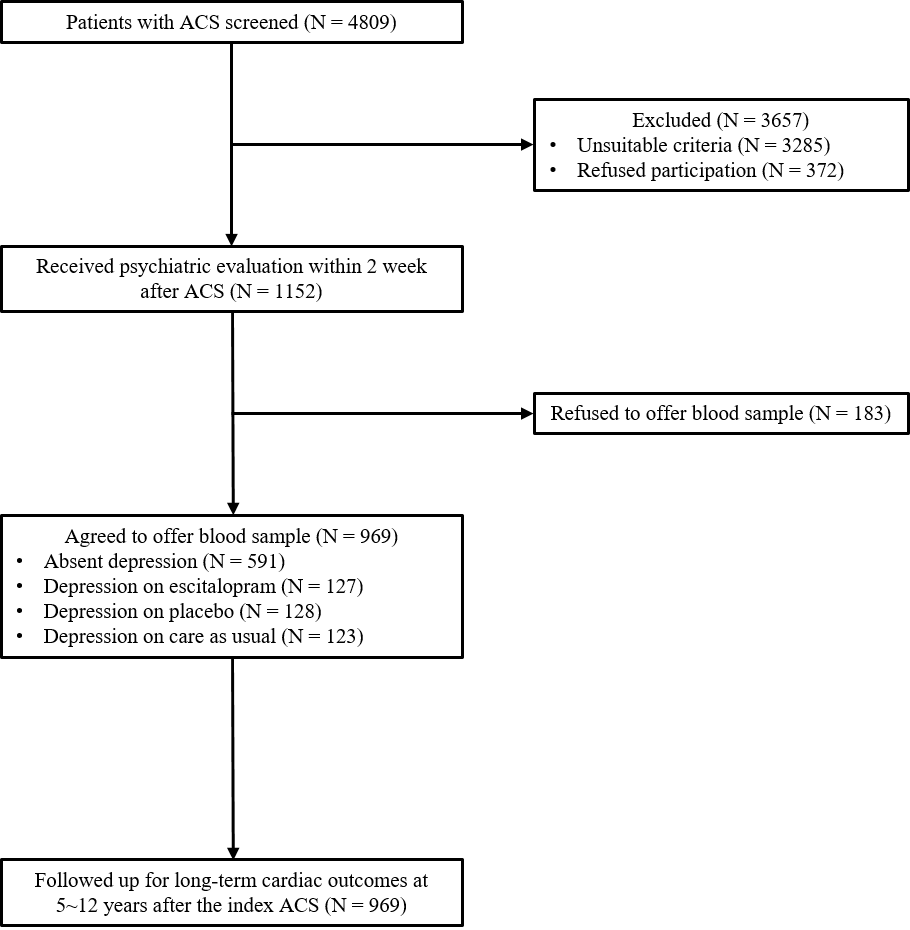


ACS: acute coronary syndrome

**Supplementary Figure 2**. Directed acylic graph of covariates.


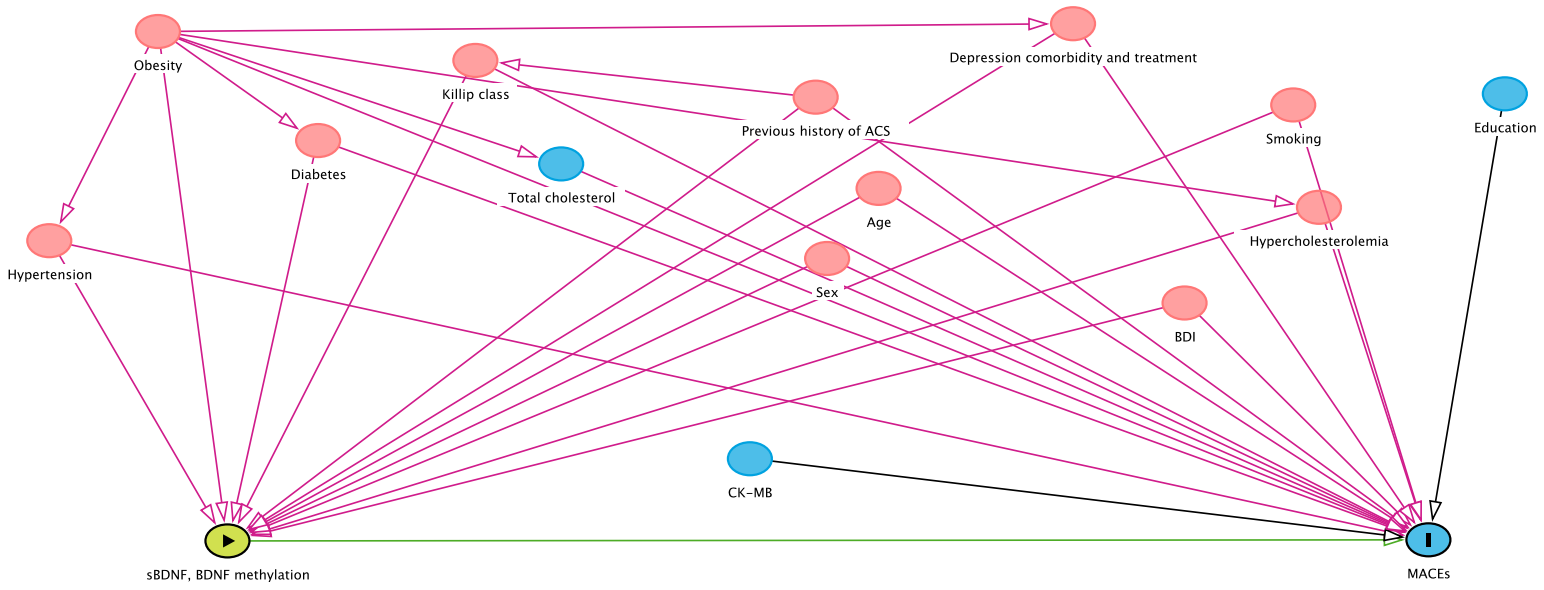


**Supplementary Figure 3**. Methylation percentages of CpG regions in *BDNF* exon VI.

Figure legends:

The CpGs are underlined and numbered. Forward and backward primers are shown, as well as sequencers. The genetic sequence is calculated from the transcriptional start site. CpG islands were determined as sequences of at least 200 bp with a GC percentage greater than 50%.


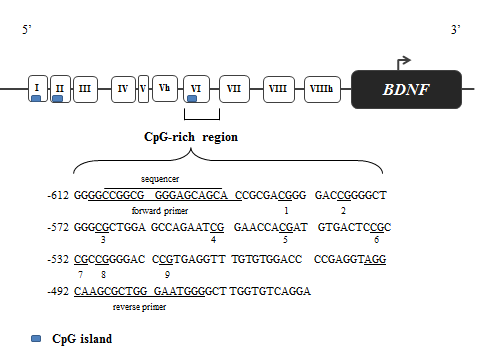


**Supplementary Figure 4**. Cumulative incidence (%) of individual major adverse cardiac events (MACEs) according to the average level of *BDNF* methylation at baseline in patients with a low sBDNF level.


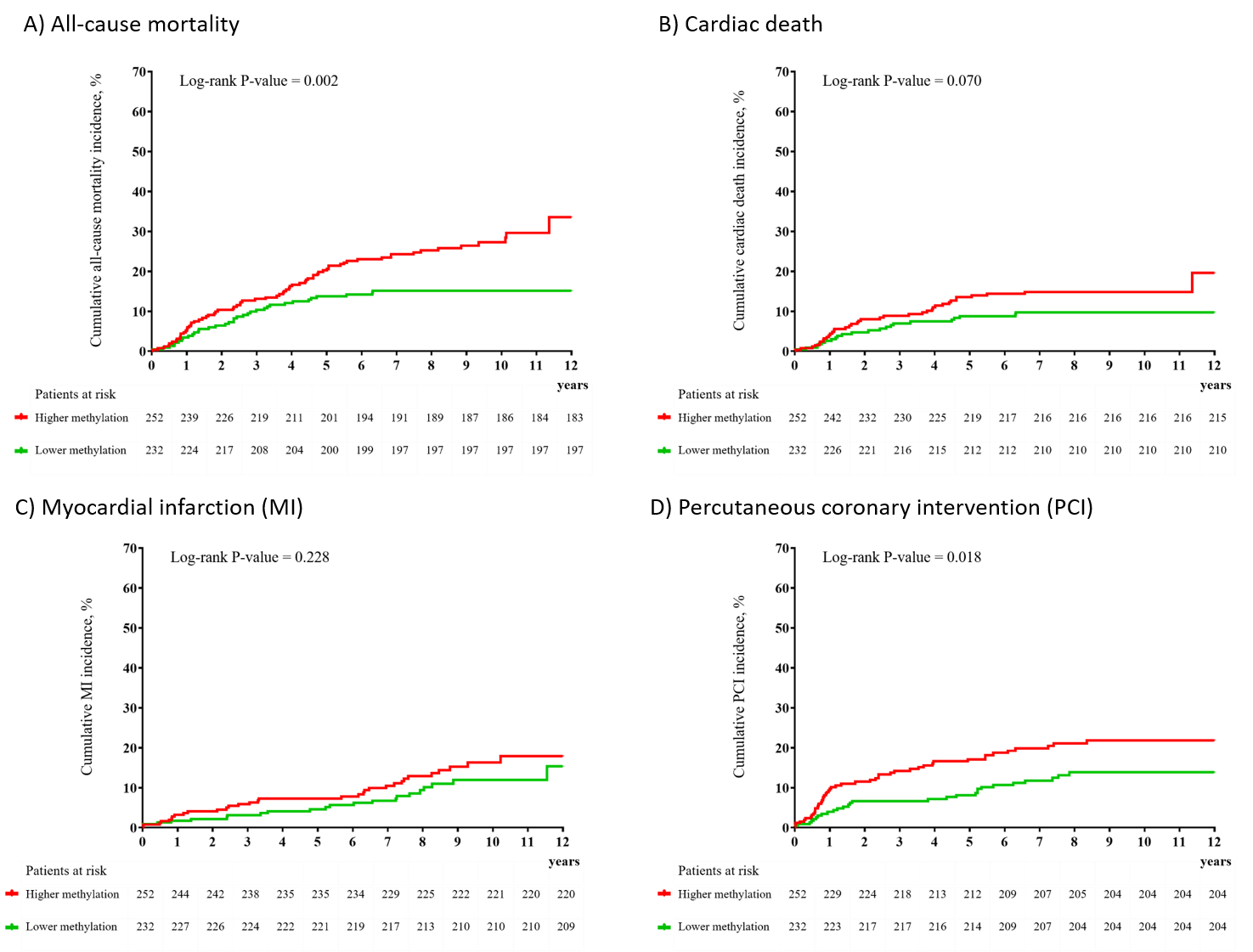


**Supplementary Figure 5**. Cumulative incidence (%) of individual MACEs according to the average level of *BDNF* methylation at baseline in patients with a high sBDNF level.


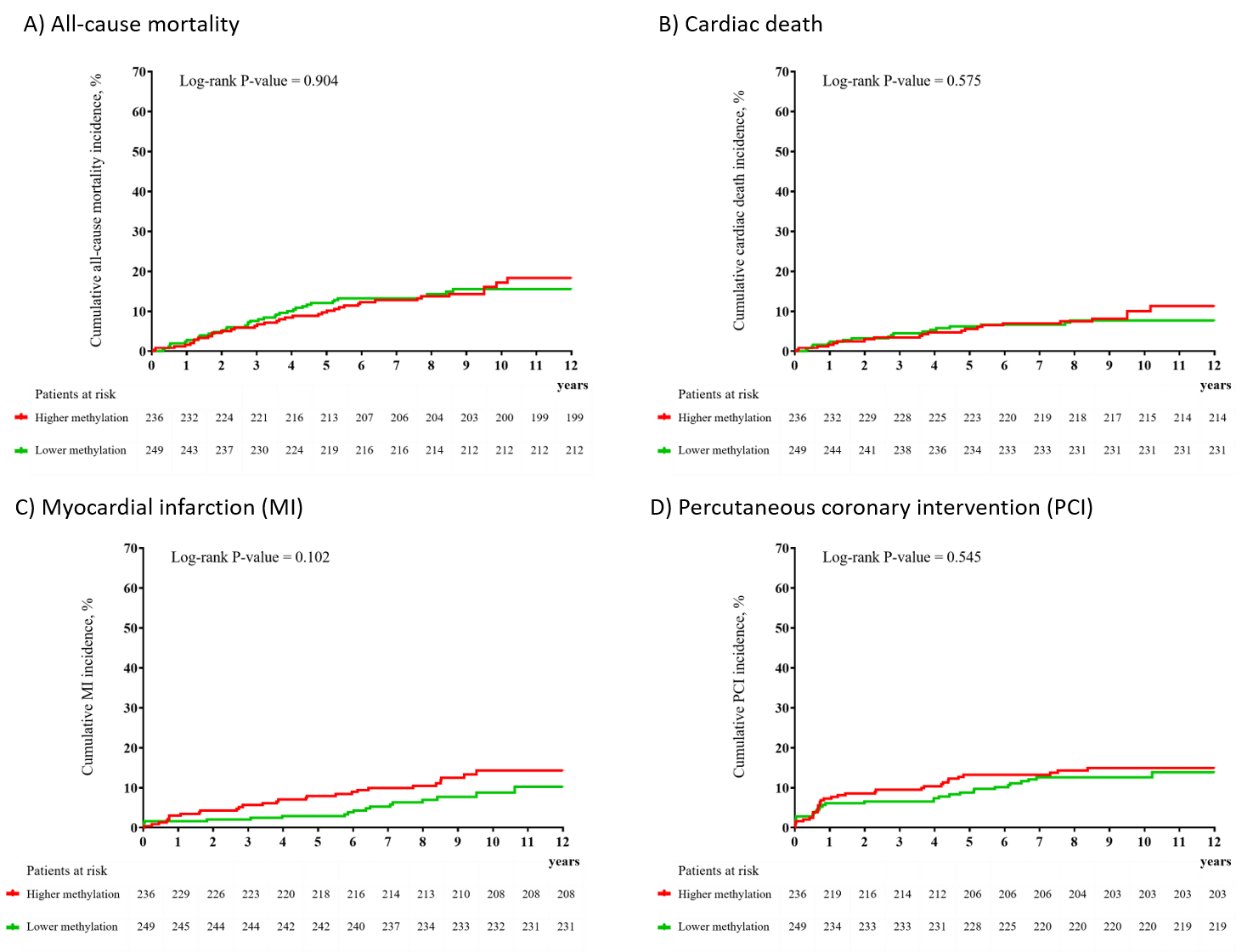


| **Supplementary Table 1.** Comparisons of serum BDNF levels and average *BDNF* methylation according to presence of the *BDNF* Val66Met polymorphism in patients with acute coronary syndrome | | | | | | |
| --- | --- | --- | --- | --- | --- | --- |
|  |  | Val/Val (N = 242) | Val/Met (N = 498) | Met/Met (N = 229) | Statistical coefficient^a^ | P-value |
| Serum BDNF (SD) ng/mL |  | 20.0 (6.8) | 17.3 (6.7)^b^ | 16.8 (7.2)^b^ | F = 15.721 | P < 0.001 |
| *BDNF* methylation (SD) % |  | 37.2 (4.7) | 38.3 (5.3)^b^ | 38.0 (5.2) | F = 3.819 | P = 0.022 |

^a^Analysis of variance (ANOVA) with post hoc Tukey’s test. ^b^P-value < 0.05 vs. Val/Val in the post hoc analysis. BDNF, brain-derived neurotrophic factor.

| **Supplementary Table 2**  Comparisons of the baseline characteristics according to the serum brain-derived neurotrophic factor (sBDNF) level in patients with acute coronary syndrome. | | | | | |
| --- | --- | --- | --- | --- | --- |
|  |  | Low sBDNF (N = 484) | High sBDNF (N = 485) | Statistical coefficient^a^ | P-value |
| **Socio-demographic characteristics** |  |  |  |  |  |
| Age, mean (SD) years |  | 59.3 (11.0) | 57.1 (11.1) | t = 3.106 | P = 0.002 |
| Sex, N (%) female |  | 136 (28.1) | 133 (27.4) | χ^2^ = 0.055 | P = 0.814 |
| Education, mean (SD) years |  | 9.7 (4.6) | 10.0 (4.7) | t = -0.946 | P = 0.344 |
| Marital status, N (%) unmarried |  | 78 (16.1) | 63 (13.0) | χ^2^ = 1.904 | P = 0.168 |
| Living alone, N (%) |  | 48 (9.9) | 44 (9.1) | χ^2^ = 0.201 | P = 0.654 |
| Housing, N (%) rented |  | 72 (14.9) | 78 (16.1) | χ^2^ = 0.269 | P = 0.604 |
| Currently unemployed, N (%) |  | 198 (40.9) | 170 (35.1) | χ^2^ = 3.529 | P = 0.060 |
| **Laboratory assessment** |  |  |  |  |  |
| Fasting glucose, mean (SD ) mg/dL |  | 136.0 (40.5) | 135.0 (47.2) | t = 0.344 | P = 0.731 |
| Total cholesterol, mean (SD) mg/dL |  | 184.7 (38.8) | 186.0 (40.2) | t = -0.505 | P = 0.614 |
| BUN, mean (SD) mg/dL |  | 15.7 (9.1) | 14.8 (10.3) | t = 1.369 | P = 0.171 |
| Creatinine, mean (SD) mg/dL |  | 0.90 (0.30) | 0.87 (0.27) | t = 1.294 | P = 0.196 |
| **Depression characteristics** |  |  |  |  |  |
| Previous depression, N (%) |  | 12 (2.5) | 22 (4.5) | χ^2^ = 3.027 | P = 0.082 |
| Family history of depression, N (%) |  | 10 (2.1) | 13 (2.7) | χ^2^ = 0.395 | P = 0.530 |
| BDI, mean (SD) score |  | 9.8 (8.7) | 10.3 (8.5) | t = -0.907 | P = 0.365 |
| Depression comorbidity and treatment, N (%) |  |  |  | χ^2^ = 1.747 | P = 0.626 |
| Absent depression |  | 296 (61.2) | 295 (60.8) |  |  |
| Depression on escitalopram |  | 59 (12.2) | 68 (14.0) |  |  |
| Depression on placebo |  | 62 (12.8) | 66 (13.6) |  |  |
| Depression on care as usual |  | 67 (13.8) | 56 (11.5) |  |  |
| **Cardiac risk factors, N (%)** |  |  |  |  |  |
| Previous ACS |  | 16 (3.3) | 23 (4.7) | χ^2^ = 1.294 | P = 0.255 |
| Family history of ACS |  | 13 (2.7) | 18 (3.7) | χ^2^ = 0.822 | P = 0.364 |
| Diabetes |  | 106 (21.9) | 85 (17.5) | χ^2^ = 2.930 | P = 0.087 |
| Hypertension |  | 238 (49.2) | 220 (45.4) | χ^2^ = 1.413 | P = 0.235 |
| Hypercholesterolemia |  | 239 (49.4) | 247 (50.9) | χ^2^ = 0.232 | P = 0.630 |
| Obesity |  | 205 (42.4) | 210 (43.3) | χ^2^ = 0.088 | P = 0.767 |
| Current smoker |  | 165 (34.1) | 201 (41.4) | χ^2^ = 5.571 | P = 0.018 |
| **Current cardiac status** |  |  |  |  |  |
| Killip class >1, N (%) |  | 101 (20.9) | 67 (13.8) | χ^2^ = 8.409 | P = 0.004 |
| LVEF, mean (SD) |  | 60.6 (11.4) | 61.8 (11.2) | t = -1.703 | P = 0.089 |
| Troponin I, mean (SD) mg/dL |  | 10.5 (15.0) | 9.3 (14.8) | t = 1.212 | P = 0.226 |
| CK-MB, mean (SD) mg/dL |  | 19.8 (44.1) | 14.9 (28.8) | t = 2.050 | P = 0.041 |

^a^Independent two-sample t-test or χ^2^ test, as appropriate. BUN, blood urea nitrogen; BDI, Beck Depression Inventory; ACS, acute coronary syndrome; LVEF, left ventricular ejection fraction; CK-MB, creatine kinase-MB.

| **Supplementary Table 3**. Associations of a higher average BDNF methylation level at baseline with long-term cardiovascular outcomes in patients with ACS, according to the sBDNF level (BDNF methylation: continuous variable). | | | |
| --- | --- | --- | --- |
|  | Low sBDNF (N = 484) | High sBDNF (N = 485) | P-value for interaction |
| Major adverse cardiac events | 1.06 (1.03-1.09)**^‡^** | 1.00 (0.98-1.02) | 0.005 |
| All-cause mortality | 1.07 (1.03-1.11)**^†^** | 1.00 (0.97-1.04) | 0.064 |
| Cardiac death | 1.06 (1.01-1.12)**^*^** | 1.01 (0.97-1.06) | 0.394 |
| Myocardial infarction | 1.01 (0.96-1.07) | 1.01 (0.97-1.06) | 0.781 |
| Percutaneous coronary intervention | 1.03 (0.99-1.08) | 0.98 (0.94-1.03) | 0.268 |

The HR (95% CI) was adjusted for age, sex, Beck Depression Inventory scores, depression comorbidity and treatment, previous history of ACS, diabetes, hypertension, hypercholesterolemia, obesity, smoking, and Killip class. **^*^**P < 0.05; **^†^**P < 0.01; **^‡^**P < 0.001.

| **Supplementary Table 4**. Interaction effects between the sBDNF (continuous variable) and average BDNF methylation (continuous variable) levels on long-term cardiovascular outcomes in patients with ACS. | |
| --- | --- |
|  | P-value for interaction |
| Major adverse cardiac events | 0.041 |
| All-cause mortality | 0.128 |
| Cardiac death | 0.470 |
| Myocardial infarction | 0.666 |
| Percutaneous coronary intervention | 0.937 |

The HR (95% CI) was adjusted for age, sex, Beck Depression Inventory scores, depression comorbidity and treatment, previous history of ACS, diabetes, hypertension, hypercholesterolemia, obesity, smoking, and Killip class.

| **Supplementary Table 5**. Hazard ratios (and 95% confidence intervals) from cause-specific hazard models for long-term cardiovascular outcome. | | | |
| --- | --- | --- | --- |
|  | Low sBDNF (N = 439) | High sBDNF (N = 450) | P-value for interaction |
| Major adverse cardiac events | 1.68 (1.21-2.32)**^†^** | 1.11 (0.78-1.60) | 0.049 |
| Cardiac death | 1.53 (0.90-2.63) | 1.34 (0.68-2.65) | 0.395 |
| Myocardial infarction | 1.16 (0.66-2.04) | 0.82 (0.34-2.00) | 0.634 |
| Percutaneous coronary intervention | 1.69 (1.05-2.72)**^*^** | 1.01 (0.59-1.71) | 0.201 |

The HR (95% CI) was adjusted for age, sex, Beck Depression Inventory scores, depression comorbidity and treatment, previous history of ACS, diabetes, hypertension, hypercholesterolemia, obesity, smoking, and Killip class. **^*^**P < 0.05; **^†^**P < 0.01.

| **Supplementary Table 6**. Hazard ratios (and 95% confidence intervals) from cause-specific hazard models for myocardial infarction and percutaneous coronary intervention. | | | |
| --- | --- | --- | --- |
|  | Low sBDNF (N = 381) | High sBDNF (N = 414) | P-value for interaction |
| Myocardial infarction | 1.18 (0.67-2.06) | 1.43 (0.75-2.70) | 0.777 |
| Percutaneous coronary intervention | 1.75 (1.09-2.82)**^*^** | 0.97 (0.57-1.65) | 0.123 |

The HR (95% CI) was adjusted for age, sex, Beck Depression Inventory scores, depression comorbidity and treatment, previous history of ACS, diabetes, hypertension, hypercholesterolemia, obesity, smoking, and Killip class. **^*^**P < 0.05.

| **Supplementary Table 7.** Effects of the sBDNF level on long-term cardiovascular outcomes in patients with ACS. | | | | | |
| --- | --- | --- | --- | --- | --- |
|  | Major adverse cardiac events | All-cause mortality | Cardiac death | Myocardial infarction | Percutaneous coronary intervention |
| High sBDNF | Reference | Reference | Reference | Reference | Reference |
| Low sBDNF | 1.23 (1.00-1.51) | 1.33 (0.98-1.80) | 1.42 (0.94-2.15) | 1.25 (0.84-1.87) | 1.15 (0.82-1.62) |

The HR (95% CI) was adjusted for age, sex, Beck Depression Inventory scores, depression comorbidity and treatment, previous history of ACS, diabetes, hypertension, hypercholesterolemia, obesity, smoking, and Killip class.

**Supplementary Methods**

**Eligibility criteria for the K-DEPACS participants**

For the K-DEPACS study entry, the inclusion criteria were as follows: i) age 18–85 years; ii) ACS confirmed by investigation (the presence of ST-segment elevation MI was defined as > 30 min of continuous chest pain, a new ST-segment elevation ≥ 2 mm on at least two contiguous electrocardiographic leads, and a CK-MB level more than three times the upper limit of normal; the presence of non-ST-segment elevation MI was diagnosed by chest pain and positive cardiac biochemical markers without new ST-segment elevation; and the presence of unstable angina was determined by chest pain within the preceding 72 h with or without ST-T wave changes or positive cardiac biochemical markers); iii) ability to complete the study questionnaires; and iv) ability to understand the study objectives and sign informed consent. The exclusion criteria were i) occurrence of ACS while hospitalized for another reason; ii) ACS developing within 3 months after a coronary artery bypass graft procedure; iii) uncontrolled hypertension (systolic blood pressure > 180 mm Hg or diastolic blood pressure > 100 mm Hg [the same criteria used in the SADHART trial]) (1); iv) resting heart rate < 40 beats/min; v) severe physical illnesses that are life threatening or interfere with ACS recovery; and vi) persistent clinically significant laboratory abnormalities in complete blood cell counts, thyroid tests, renal function tests, or liver function tests.

***BDNF* methylation**

These data have been deposited in GenBank (accession number: BankIt1568919 BDNF JX848620). The *BDNF* region evaluated for methylation was chosen because it corresponds to an analogous region of rat *BDNF* that is differentially methylated and associated with BDNF mRNA expression, and this region has been investigated in the context of antenatal depression (2, 3). Genomic DNA (1 μg) was extracted from leukocytes using the QIAamp DNA Blood Mini Kit (Qiagen, Valencia, CA, USA). The DNA was then subjected to bisulfite treatment using the EpiTech Bisulfite Kit (Qiagen). A 150 bp fragment of the *BDNF* promoter was amplified by PCR from bisulfite-treated DNA using the forward (5′-GTGGGGTAGGAGGGGAGTAGTAT-3′) and reverse (5′-AAATCCCCCAATCAACTCTCT-3′) primers. PCR conditions were as follows: 95°C for 15 min, followed by 45 cycles of 95°C for 15 s, 57°C for 30 s, and 72°C for 15 s, with a final extension at 72°C for 5 min. The PCR products were sequenced using the PSQ 96M Pyrosequencing System (Biotage,Uppsala, Sweden) with the primer 5′-GGTAGGAGGGGAGTAGTA-3′. The methylation percentage at each CpG region was quantified using Pyro Q-CpG software, version 1.0.9 (Biotage,Uppsala, Sweden). The average methylation percentages at the six CpG sites were used in the analyses. Because the methylation percentages at CpG sites 2, 6, and 8 were 100% in all participants, these three sites were excluded.

**Long-term follow-up for cardiovascular outcomes**

After patients were enrolled in this study, long-term follow-up was started. Every time a patient visited the hospital, KAMIR researchers delivered the KAMIR protocol and evaluated and recorded each patient’s condition. In order to collect precise information on long-term cardiovascular outcomes, at least 2 researchers were dedicated fully to this investigation for the course of the study. These researchers called patients or their family members the day before each anticipated hospital visit to encourage continuous participation and to maximize follow-up. The KAMIR database, which is shared throughout hospitals, and a phone call to the hospital were both used by the researchers to confirm patient’s condition for those who had been transferred to other institutions. When causes of loss to follow-up could not be found in the hospital records, mainly deaths outside of the hospital, deaths were confirmed through phone contact with a family member and through death certification in the National Registration Records.

**References**

1. Glassman AH, O'Connor CM, Califf RM, Swedberg K, Schwartz P, Bigger JT, Jr., et al. Sertraline Treatment of Major Depression in Patients with Acute Mi or Unstable Angina. *JAMA* (2002) 288(6):701-9. Epub 2002/08/10. doi: 10.1001/jama.288.6.701.

2. Devlin AM, Brain U, Austin J, Oberlander TF. Prenatal Exposure to Maternal Depressed Mood and the Mthfr C677t Variant Affect Slc6a4 Methylation in Infants at Birth. *PLoS One* (2010) 5(8):e12201. Epub 2010/09/03. doi: 10.1371/journal.pone.0012201.

3. Roth TL, Lubin FD, Funk AJ, Sweatt JD. Lasting Epigenetic Influence of Early-Life Adversity on the Bdnf Gene. *Biol Psychiatry* (2009) 65(9):760-9. Epub 2009/01/20. doi: 10.1016/j.biopsych.2008.11.028.
